# Five-Year Outcomes of Patients With Mitral Structural Valve Deterioration Treated With Transcatheter Valve in Valve Implantation – A Single Center Prospective Registry

**DOI:** 10.3389/fcvm.2022.883242

**Published:** 2022-04-26

**Authors:** Nili Schamroth Pravda, Raffael Mishaev, Amos Levi, Guy Witberg, Yaron Shapira, Katia Orvin, Yeela Talmor Barkan, Ashraf Hamdan, Ram Sharoni, Leor Perl, Alexander Sagie, Hana Vaknin Assa, Ran Kornowski, Pablo Codner

**Affiliations:** ^1^Department of Cardiology, Rabin Medical Center, Petah Tikva, Israel associated with Faculty of Medicine, Tel Aviv University, Tel Aviv, Israel; ^2^Sackler Faculty of Medicine, Tel Aviv University, Tel Aviv, Israel; ^3^Cardio-Thoracic Surgery Department, Faculty of Medicine, Rabin Medical Center, Tel Aviv University, Tel Aviv, Israel

**Keywords:** mitral valve, structural valve deterioration, valve-in-valve, transcatheter, outcomes

## Abstract

The Valve-in-Valve (ViV) technique is an emerging alternative for the treatment of bioprosthetic structural valve deterioration (SVD) in the mitral position. We report on intermediate-term outcomes of patients with symptomatic SVD in the mitral position who were treated by transcatheter mitral valve-in-valve (TM-ViV) implantation during the years 2010–2019 in our center. Three main outcomes were examined during the follow-up period: NYHA functional class, TM-ViV hemodynamic data per echocardiography, and mortality. Our cohort consisted of 49 patients (mean age 77.4 ± 10.5 years, 65.3% female). The indications for TM-ViV were mainly for regurgitant pathology (77.6%). All 49 patients were treated with a balloon-expandable device. The procedure was performed *via* transapical access in 17 cases (34.7%) and transfemoral vein/trans-atrial septal puncture in 32 cases (65.3%). Mean follow-up was 4.4 ± 2.0 years. 98% and 91% of patients were in NYHA I/II at 1 and 5 years, respectively. Mitral regurgitation was ≥moderate in 86.3% of patients prior to the procedure and this decreased to 0% (*p* < 0.001) following the procedure and was maintained over 2 years follow-up. The mean trans-mitral valve gradients decreased from pre-procedural values of 10.1 ± 5.1 mmHg to 7.0 ± 2.4 mmHg at 1 month following the procedure (*p* = 0.03). Mortality at 1 year was 16% (95%, CI 5–26) and 35% (95%, CI 18–49) at 5 years. ViV in the mitral position offers an effective and durable treatment option for patients with SVD at high surgical risk.

## Introduction

Bioprosthetic surgical valve replacement for the treatment of native valve disease has increased over the last two decades, resulting in an increased number of patients presenting with structural valve deterioration (SVD). The treatment of failed bioprosthetic valves has traditionally been surgical valve replacement. However, in those at increased surgical risk, reoperation has associated substantial morbidity and mortality (1, 2). Trans-catheter mitral valve-in-valve (TM-ViV) implantation inside failed surgically implanted bio-prostheses is an increasingly used, less invasive, alternative to repeat surgery in high-risk patients (3, 4). We report on our clinical experience of treating patients in the mitral position using the ViV technique in our institution, aiming to provide insights into the intermediate-term clinical outcomes of these patients.

## Materials and Methods

The characteristics and outcomes of patients with bioprosthetic SVD treated by the implantation of a TM-ViV device within a failed surgical valve are described in the present report. The cohort included patients undergoing TM-ViV procedures performed from October 2010 to October 2019. Patient data follow-up was completed until November 2021. Operative risk was determined by the logistic European System for Cardiac Operative Risk Evaluation score (log EUROSCORE) and the Society of Thoracic Surgeons (STS) score (5, 6). All patients were discussed in the setting of local Heart Team with interventional and imaging cardiologists and cardio-thoracic surgeon with experience in mitral valve surgery. All patients underwent transthoracic and transesophageal (TEE) echocardiogram. The baseline, procedural and peri-procedural findings are described. The prospective data collection was approved by the institutional review board. Three endpoints were examined: NYHA (New York Heath Association) functional status at 1- and 5-year, valve hemodynamic of the implanted valves as per echocardiography done at 1 month after the procedure and yearly thereafter and rates of survival during the follow-up period.

Data on mortality was based on mortality files derived from the notification of death form legally required by the Ministry of the Interior. Subgroup analysis was done comparing mortality of those undergoing the procedure transapically vs. trans atrial-septal approach. Follow-up data were available for 42 patients at 1-year follow-up and 11 patients at 5-year follow-up.

Baseline characteristics of the patients are presented as mean and standard deviation (SD) for continuous variables and count (%) for categorical variables. Continuous variables were compared using the Student’s *t*-test/Mann–Whitney U test, categorical variables were compared using the chi-square/Fisher’s exact test, as appropriate. All tests were 2 tailed, and a *p*-value <0.05 was considered significant. All-cause mortality was graphically plotted using Kaplan–Meier curves and compared between groups using the log rank test (unadjusted analysis). All TM-ViV-related data was registered in an electronic file and analyzed using the SPSS, version 25.0, software (SPSS, Chicago, IL, United States).

## Results

The baseline clinical and echocardiographic characteristics of the cohort are shown in Tables 1, 2. The cohort consisted of 49 patients with a mean age of 77.4 ± 10.5 years, female 65.3%. The mean STS score was 7.7 ± 6.5 and most patients (78%) were in NYHA (New York Heart Association) functional class III/IV at baseline. The average time to TM-ViV from surgical mitral valve replacement was 11.3 ± 3.7 years. The indication for TM-ViV was predominantly for regurgitant pathology (77.6%). Most patients (95.9%) had normal left ventricular function (Ejection Fraction ≥50%) at baseline. Average follow up was 4.4 ± 2.0 years following the procedure.

**TABLE 1:** Baseline characteristics of the cohort.

TM-ViV	*N* = 49 (%)
Age (years)	77.4 ± 10.5
Male (%)	17 (34.7)
BMI (kg/m^2^)	27.1 ± 5.1
STS score	7.7 ± 6.5
Euroscore II	8.9 ± 4.3
Coronary artery disease (%)	16 (32.7)
Prior coronary artery bypass surgery (%)	14 (35.0)
Prior PCI (%)	5 (12.5)
Prior CVA/TIA (%)	7 (14.6)
Peripheral vascular disease (%)	4 (8.2)
Diabetes mellitus (%)	11 (22.9)
Hypertension (%)	42 (87.5)
Chronic dialysis (%)	1 (7.7)
Chronic obstructive pulmonary disease (%)	12 (24.5)
Atrial fibrillation/flutter (%)	34 (69.4)
NYHA functional class III/IV (%)	36 (75.0)
Permanent pacemaker/defibrillator (%)	5 (10.2)
Hemoglobin (g/dL)	11.5 ± 1.7
GFR (MDRD) (mL/Min/m^2^)	55.9 ± 26.8
Albumin (g/dL)	3.9 ± 0.5

*BMI, Body mass index; STS, Society of Thoracic Surgeons; PCI, Percutaneous Coronary Intervention; CVA/TIA, Cerebrovascular Accident/Transient Ischemic Attack; NYHA, New York Heart Association; GFR, glomerular Filtration rate; MDRD, Modification of Diet in Renal Disease.*

**TABLE 2:** Baseline echocardiographic characteristics.

TM-ViV	*N* = 49 (%)
Valve pathology	
Stenosis (%)	7 (14.3)
Regurgitation (%)	38 (77.6)
Combined (%)	4 (8.2)
Peak gradient (mmHg)	20.7 ± 9.0
Mean gradient (mmHg)	10.1 ± 5.1
LVEF (%) (>50%)	47 (95.9)
Mean systolic pulmonary artery pressure (mmHg)	50 mmHg (IQR 47.5,75)
**Size of valve treated (mm)**	
25	5 (10.2)
27	24 (48.9)
29	10 (20.4)
31	8 (16.3)
33	2 (4.0)

*TM-ViV, Transcatheter Mitral Valve-in-Valve; LVEF = Left Ventricular Ejection Fraction.*

### Procedural Characteristics

Procedural characteristics are shown in Table 3. From October 2010 to January 2017 the procedures were performed *via* transapical access (17 cases, 34.7%). From January 2017 onward, procedures were done *via* a transfemoral vein and trans-atrial septal puncture (32 cases, 65.3%). All 49 patients were treated with Sapien XT™ (*n* = 17) or Sapien™ 3 (*n* = 32) (Edwards Lifesciences, Irvine, CA, United States), balloon-expandable, transcatheter heart valves. All cases were performed under intra-procedural TEE guidance to assist during trans-septal puncture, assess valve hemodynamics, left ventricular outflow tract (LVOT) obstruction and to assess for significant shunt throughout the iatrogenic atrial septal defect. There were 4 cases in which an iatrogenic atrial septal defect from transseptal balloon septostomy was closed following the TM-ViV procedure, 3 of which were done during the index procedure. There were no cases of acute LVOT obstruction in our cohort. The average hospital stay was 5.9 ± 4.8 days. There were no events of periprocedural strokes. There were 2 patients who needed post-procedural permanent pacemaker insertion. The list of bioprosthetic valves type and size and their corresponding transcatheter valve device is shown in appendix (Supplementary Table 1).

**TABLE 3:** Procedural characteristics.

TM-VIV	*N* = 49 (%)
Procedure urgent (%)	10 (20.4)
Fluoroscopy time (min)	25.4 ± 18.0
Contrast volume (ml)	24.0 ± 39.1
General anesthesia	49 (100)
**Access (%)**	
Transapical	17 (34.7)
Femoral vein/trans-atrial septal	32 (65.3)
**TM VIV size (mm)**	
26	32 (65.3)
29	17 (34.7)
Concomitant PCI (%)	3 (6.1)
Concomitant paravalvular leak closure (%)	2 (4.1)
Balloon expandable valve (%)	49 (100)
Concomitant Iatrogenic atrial septal defect closure (%)	4 (8.2)

*TM-ViV, Transcatheter Mitral Valve-in-Valve; PCI, Percutaneous Coronary Intervention.*

### Functional Status

**FIGURE 1:**
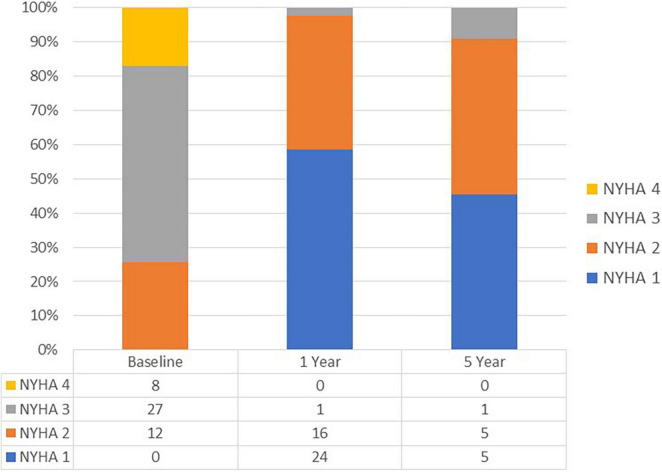
NYHA functional class during follow up.

As shown in Figure 1 at 1 year, data was available for 41 patients, of which 98% (*n* = 40) were in NYHA functional class I/II at 1 year. At 5-year follow up, data was available for 11 patients of which 91% (*n* = 10) were in NYHA I/II.

### Hemodynamic Parameters

**FIGURE 2:**
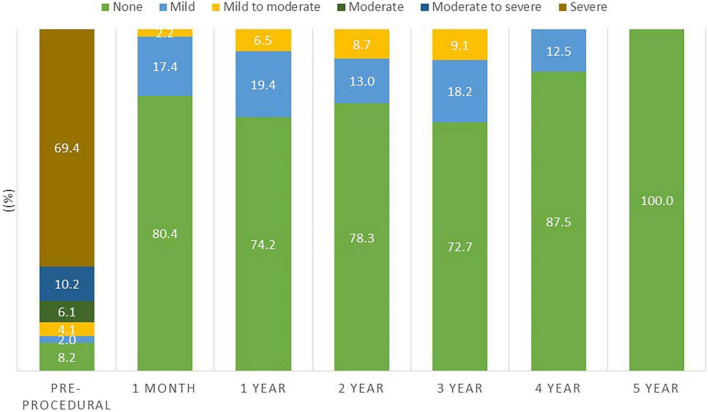
Degree of mitral regurgitation during follow up.

**FIGURE 3:**
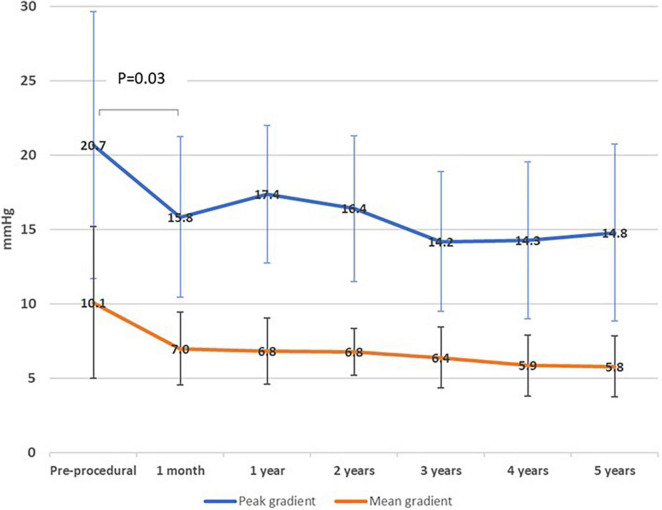
Mitral valve gradients during follow up.

The temporal changes in hemodynamic indexes were assessed by echocardiography. As shown in Figure 2, mitral regurgitation was ≥moderate in 85.6% of patients prior to the procedure and this decreased to 0% (*P* < 0.001) following the procedure (1 month) and was maintained over 5 years follow up. The mean peak and mean trans-mitral valve gradients decreased from pre-procedural values of 20.7 ± 9.0 mmHg and 10.1 ± 5.1 mmHg, respectively, to 15.8 ± 5.4 and 7.0 ± 2.4 mmHg at 1 month following the procedure, respectively, *p* = 0.03 (Figure 3). The values for peak and mean mitral valve gradient remained reduced during the follow up period. Systolic Pulmonary artery pressures decreased from 50 mmHg (IQR 47.5,75) to 47 mmHg (IQR35.5,58) at 1 month follow up, *p* = 0.016 (Supplementary Figure 1). Tricuspid regurgitation was ≥moderate in 41% of the cohort prior to the procedure and this did not decrease significantly following the procedure (Supplementary Figure 2).

### Mortality and Major Complications

**FIGURE 4:**
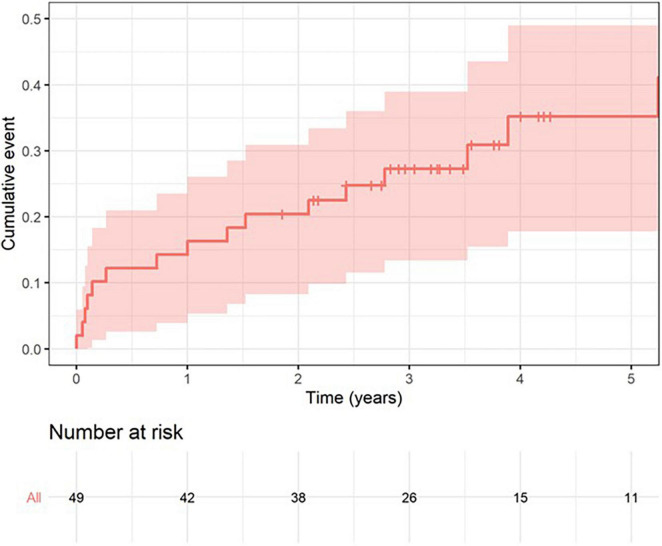
Mortality during follow up.

Mortality at 1 year and at 5 years was 16% (95% CI 5–26) and 35% (95% CI 18–49), respectively (Figure 4). In a sub-group analysis, there were no differences in mortality between patients that underwent the procedure *via* transapical or transfemoral/transeptal access (Supplementary Figure 3). There were 3 events of in-hospital death. The first was in a patient who had a concomitant percutaneous mitral paravalvular leak closure using an Amplazer device. This patient’s hospitalization was complicated by severe hemolysis, acute-on-chronic kidney injury, sepsis, and ultimate demise. The second patient died from myocardial rupture during TM-ViV insertion, consequent tamponade, and death. The third was a case of hemorrhagic shock due to vascular complications during the procedure. There were two cases of infective endocarditis, both more than 1 year following the procedure [methicillin-sensitive Staphylococcus aureus (MSSA) and coagulase-negative staphylococci (CONS)] and one case of an ischemic stroke in a patient 3 months following the procedure who had concomitant atrial fibrillation.

## Discussion

The main objective of our study was to report on the intermediate-term clinical outcomes of patients with SVD treated with TM-ViV from our all-comer single center experience.

Our findings from this retrospective study demonstrated the following: firstly, the functional NYHA status of vast majority of the cohort significantly improved following TM-ViV and this clinical improvement was maintained at follow-up. Secondly, the transvalvular gradients and hemodynamic response to the procedure were favorable among patients and maintained over the duration of the follow-up period. Lastly, and most importantly, all-cause mortality at 1-year and 5-year follow-ups were relatively low, with mortality rates of 16% (95% CI 5–26) and 35% (95% CI 18–49), respectively.

Our findings are congruent with previous reports showing significant improvement in functional status following TM-ViV implantation (4, 7). Okoh et al. reported that 80% of surviving patients were at NYHA I/II at 30-day and 1-year follow-up. While these results show an overall high success rate, we expand on these findings showing 98% of surviving patients were at NYHA I/II at 1-year. Moreover, the improvement in functional status was maintained at 5-year follow-up in 91% of surviving patients. Our analysis of valve hemodynamics by echocardiography were consistent with previously reported studies (8, 9). Mitral regurgitation (MR) severity post-procedure decreased significantly from 86.3 to 0% (*p* < 0.001) and this was maintained over follow-up. The mean peak and mean trans-mitral valve gradients decreased significantly following the procedure but remained mildly elevated – however, this did not hinder clinical improvement as reflected by the marked improvement in NYHA functional class. Whisenant et al. reported similar findings in their cohort, with a marked improved in functional status even though the mean mitral valve gradients were on average 7.3 ± 2.73 mmHg at 30 days follow up (10). The TM-ViV procedure adds a valve into the already limited effective orifice area of the previous mitral valve bioprosthesis and there remains an element of mitral stenosis secondary to the procedure which can explain the valve hemodynamics. The pulmonary artery pressures did not decrease significantly – the pulmonary hypertension secondary to mitral valve disease can often be irreversible due to its chronicity and structural changes in the pulmonary vasculature itself and remain elevated despite an adequate structural intervention (11). It is important to note that while the valve hemodynamics improved significantly, there was a lack of improvement in those with concomitant tricuspid regurgitation. Secondary tricuspid regurgitation and pulmonary pressures can be irreversible following the procedure.

In our study, the mortality rates at 1 and 5 years were 16 and 35%, respectively. Whisenant et al. reported in 2020 in their large study of 1,529 patients that their 1-year mortality rate was 16.7% (10). These improvements in clinical outcomes reflects a better case selection process, increased operator experience and a refinement of the procedural technique over the years (9, 10). Although in our sub-group analysis there were no differences in mortality between patients that underwent the procedure *via* transapical or transfemoral/transeptal access, Whisenant et al. reported a lower 1-year all-cause mortality in patients treated transeptally vs. transapically (15.8% vs. 21.7%, *P* = 0.03) (10). Zubarevich et al. report 1-year and 3-year mortalities of 28 and 37%, respectively in patients treated solely with transapical access (3). These differences may be explained by the fact that transeptal procedures are minimally invasive and therefore avoid thoracic surgical interventions, leading to faster patient recovery, less peri-procedural complications, and shorter hospital stays. Our findings should be interpreted with caution as our cohort was small.

Transcatheter mitral valve-in-valve procedures *via* transeptal access require puncture through the atrial septum, and inter-atrial balloon septostomy, leading to iatrogenic atrial septal defects (iASDs) which can often have hemodynamic consequences (12). There are currently no guidelines as to which iASDs should be closed and therefore necessitates the need for more research. iASD can be advantageous in patients with elevation left atrial pressure as they allow pressure unloading *via* a left to right shunt. In our series, there were 4 cases of TM-ViV that underwent subsequent iASD closure. These cases were performed as the iASD shunt was deemed significant clinically, mainly due right-to-left shunt, causing systemic hypoxemia.

There is increasing evidence that the percutaneous route may be advantageous over repeat mitral valve surgery. Khan et al. recently published a retrospective comparison between patients undergoing TM-ViV and re-do surgery (13). They found that TM-ViV was associated with a significantly better survival, significantly less periprocedural complications, shorter hospital stays and cost. This was despite the older age and higher burden of comorbidities amongst those undergoing TM-ViV. This advantageous slant to TM-ViV is most likely multifactorial. A pivot factor is that with TM-ViV, the valve in placed within the existing frame of the previous bioprosthesis. While with surgery, the tissue within the mitral valve apparatus is manipulated and this can negatively affect left ventricular function (14, 15). Redo surgical mitral valve replacement carries significant morbidity and mortality (9). However, more data is needed regarding the durability and long-term outcomes of redo surgery vs. TM-ViV. TM-ViV has emerged as a relatively safe and effective treatment for patients suffering from SVD. However, unlike for aortic valve replacement treatment, there are no homogenous criteria and guidelines to report outcomes for research purposes. The Valve Academic Research Consortium (VARC) was founded to standardize definitions of outcomes and endpoints after aortic valve replacement, leading to the rapid development of novel therapies and advancements in clinical research (16). There is an increasing need for standardized criteria for TM-ViV, yet no such consortium exists for mitral valve replacements. Furthermore, randomized control studies of larger cohorts are necessary to validates our findings of safety, efficacy, and durability of TM-ViV implantations.

As the number of patients undergoing TM-ViV increases, so does the incidence of post-procedural complications. The concept of neo LVOT obstruction has been documented in previous reports of TM-ViV complications (10, 17). The use of preprocedural computed tomography (CT) imaging has been shown to be essential in the prevention of this complications (17). We reported no cases of significant LVOT obstruction in our study. CT has become a routine imaging tool in the pre-procedure assessment of our patients. The risk of LVOT obstruction in our patient population was assessed and discussed with careful analysis of the available imaging using predicted LVOT measurement, assessing the length of the prosthetic surgical valve leaflets, measuring the dimensions of the LVOT, assessing for bulging intraventricular septum and planning the TM-ViV height of implantation accordingly.

The main strengths of our study are the quality of our data acquisition and the intermediate-term follow-up. We are a center with a dedicated structural intervention team with increasing experience and data spanning almost 10 years. We have a dedicated data collection team and structured clinical and imaging follow-up program to ensure careful data acquisition and quality. The TM-ViV procedure offers a safe, feasible, less invasive, and long-lasting solution to structural valve deterioration. We report longer follow-ups than previous papers and show an increase in functional status, improvement in structural hemodynamics, and decrease in mortality 5-years post-procedure.

Study limitations are that this is a single-center retrospective analysis and a relatively small cohort. There is inherent selection bias of our cohort as all patients underwent a thorough assessment process as candidates for this procedure. This is evident as the average STS score was intermediate (and not very high-risk) and that most patients had left ventricular ejection fraction about 50%. These factors may partially explain our encouraging outcomes.

## Conclusion

Transcatheter mitral valve-in-valve for the treatment of SVD yielded encouraging results in safety and efficiency in our single-center experience. Our cohort had significant clinical and hemodynamical improvement with promising intermediate-term results. TM-ViV is an emerging treatment option for SVD in the mitral position.

## Data Availability Statement

The original contributions presented in the study are included in the article/Supplementary Material, further inquiries can be directed to the corresponding author/s.

## Ethics Statement

The studies involving human participants were reviewed and approved by Helsinki Ethics Committee Rabin Medical Center. Written informed consent for participation was not required for this study in accordance with the national legislation and the institutional requirements.

## Author Contributions

All authors contributed substantial contributions to the conception or design of the work, or the acquisition, analysis or interpretation of data for the work, including drafting the work or revising it critically for important intellectual content, provide approval for publication of the content, and agree to be accountable for all aspects of the work in ensuring that questions related to the accuracy or integrity of any part of the work are appropriately investigated and resolved.

## Conflict of Interest

The authors declare that the research was conducted in the absence of any commercial or financial relationships that could be construed as a potential conflict of interest.

## Publisher’s Note

All claims expressed in this article are solely those of the authors and do not necessarily represent those of their affiliated organizations, or those of the publisher, the editors and the reviewers. Any product that may be evaluated in this article, or claim that may be made by its manufacturer, is not guaranteed or endorsed by the publisher.

## Supplementary Material

Supplementary Figure 1 Systolic pulmonary artery pressures during follow up.

Supplementary Figure 2 Degree of tricuspid regurgitation during follow up.

Supplementary Figure 3 Mortality as per transapical vs. transvenous/transeptal approach.
