# Supplementary figures and images for: Five-Year Outcomes of Patients With Mitral Structural Valve Deterioration Treated With Transcatheter Valve in Valve Implantation – A Single Center Prospective Registry
Source: Front Cardiovasc Med. 2022 Apr 26;9:883242. doi: 10.3389/fcvm.2022.883242 (PMC9086553; doi:10.3389/fcvm.2022.883242)

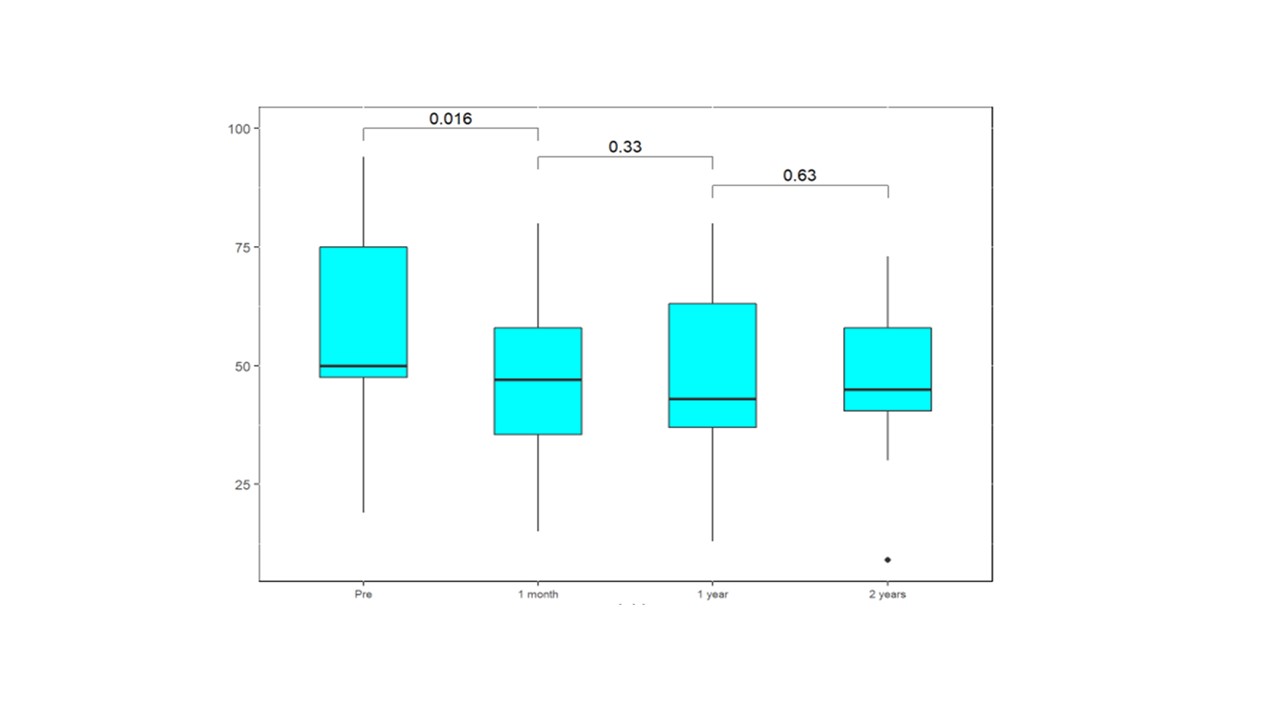

Supplement: Supplementary Figure 1 — Systolic pulmonary artery pressures during follow up. [file Image_1.JPEG]

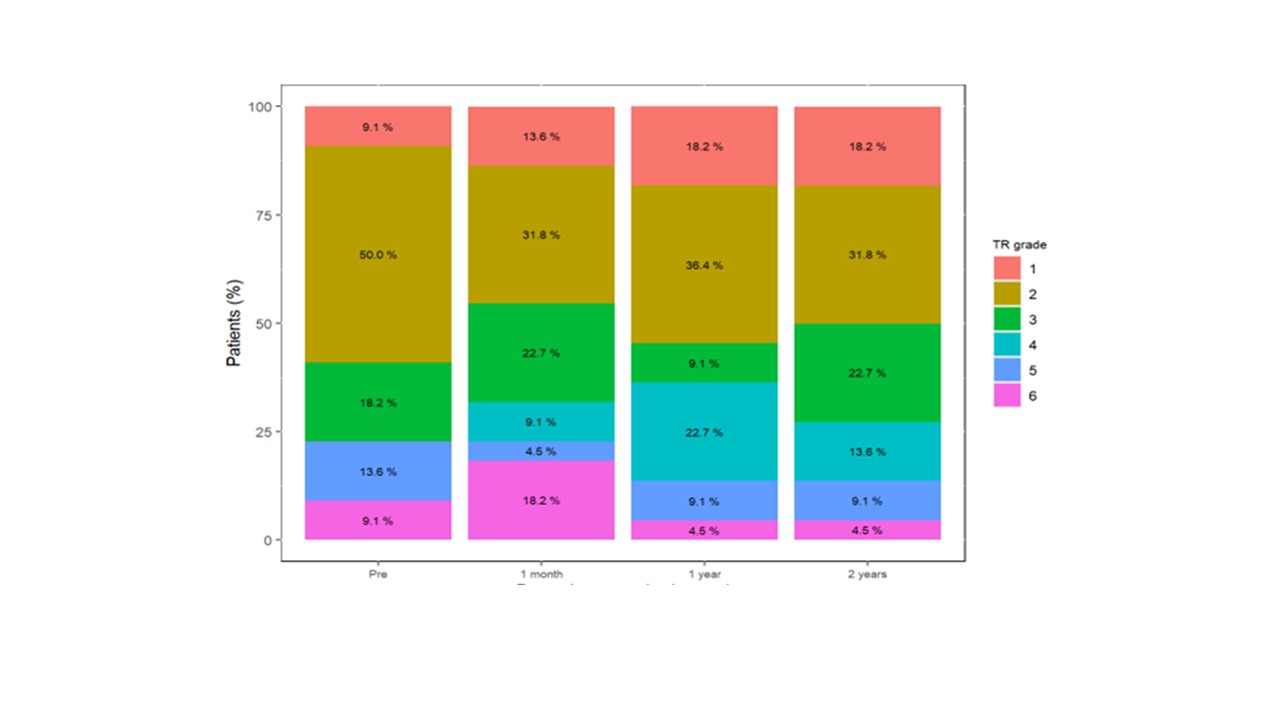

Supplement: Supplementary Figure 2 — Degree of tricuspid regurgitation during follow up. [file Image_2.JPEG]

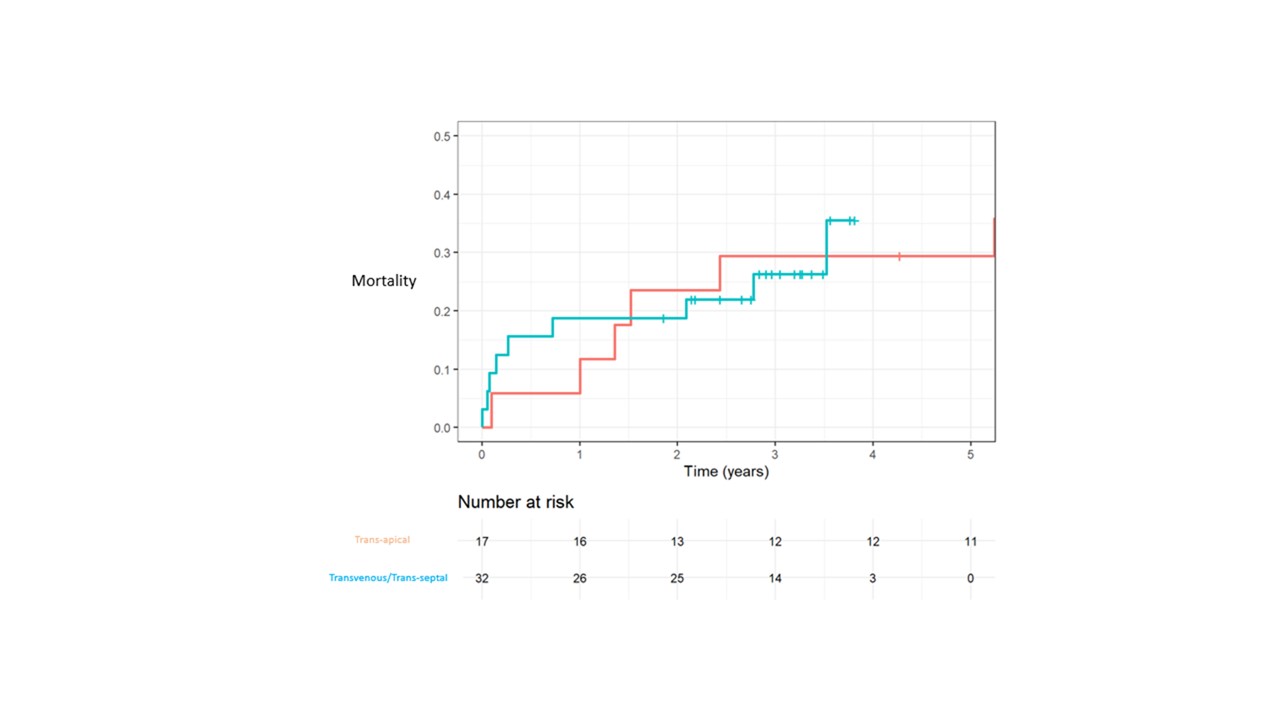

Supplement: Supplementary Figure 3 — Mortality as per transapical vs. transvenous/transeptal approach. [file Image_3.JPEG]
