# Supplementary material for: Five-Year Outcomes of Patients With Mitral Structural Valve Deterioration Treated With Transcatheter Valve in Valve Implantation – A Single Center Prospective Registry
Source: Front Cardiovasc Med. 2022 Apr 26;9:883242. doi: 10.3389/fcvm.2022.883242 (PMC9086553; doi:10.3389/fcvm.2022.883242)
Supplement: Supplementary file 4 [file Table_1.DOCX]

**Supplementary Table 1**

| Tradename Surgical Valve | Device |
| --- | --- |
| 1. Carpentier Edwards 27 | Edwards Sapien XT 26 |
| 1. Carpentier Edwards 25 | Edwards Sapien XT 26 |
| 1. Carpentier Edwards 27 | Edwards Sapien XT 26 |
| 1. Magna 25 | Edwards Sapien XT 26 |
| 1. Xenograft 31 | Edwards Sapien XT 29 |
| 1. Hancock II 27 | Edwards Sapien XT 26 |
| 1. Carpentier Edwards 27 | Edwards Sapien XT 26 |
| 1. Hancock II 29 | Edwards Sapien XT 29 |
| 1. Hancock II 29 | Edwards Sapien XT 29 |
| 1. Carpentier Edwards 31 | Edwards Sapien XT 29 |
| 1. Carpentier Edwards 27 | Edwards Sapien XT 26 |
| 1. Xenograft 27 | Edwards Sapien XT 26 |
| 1. Hancock II 27 | Edwards Sapien XT 26 |
| 1. Carpentier Edwards 27 | Edwards Sapien XT 26 |
| 1. Carpentier Edwards 31 | Edwards Sapien XT 29 |
| 1. Hancock II 27 | Edwards Sapien XT 26 |
| 1. Hancock II 27 | Edwards Sapien XT 26 |
| 1. Carpentier Edwards 27 | Edwards Sapien 3 26 |
| 1. Hancock II 33 | Edwards Sapien 3 29 |
| 1. Carpentier Edwards 27 | Edwards Sapien 3 26 |
| 1. Carpentier Edwards 29 | Edwards Sapien 3 29 |
| 1. Xenograft 31 | Edwards Sapien 3 29 |
| 1. Hancock II 27 | Edwards Sapien 3 26 |
| 1. Mosaic 25 | Edwards Sapien 3 26 |
| 1. Carpentier Edwards 31 | Edwards Sapien 3 29 |
| 1. Carpentier Edwards 25 | Edwards Sapien 3 26 |
| 1. Carpentier Edwards 27 | Edwards Sapien 3 26 |
| 1. Carpentier Edwards 27 | Edwards Sapien 3 26 |
| 1. Carpentier Edwards 27 | Edwards Sapien 3 26 |
| 1. Hancock II 27 | Edwards Sapien 3 26 |
| 1. Hancock II 27 | Edwards Sapien 3 26 |
| 1. Carpentier Edwards 29 | Edwards Sapien 3 29 |
| 1. Mosaic 27 | Edwards Sapien 3 26 |
| 1. Carpentier Edwards 31 | Edwards Sapien 3 29 |
| 1. Mosaic 29 | Edwards Sapien 3 29 |
| 1. Hancock II 27 | Edwards Sapien 3 26 |
| 1. Magna 25 | Edwards Sapien 3 26 |
| 1. Hancock II 29 | Edwards Sapien 3 29 |
| 1. Mosaic 29 | Edwards Sapien 3 26 |
| 1. Hancock II 27 | Edwards Sapien 3 26 |
| 1. Mosaic 27 | Edwards Sapien 3 26 |
| 1. Epic 31 | Edwards Sapien 3 29 |
| 1. Hancock II 29 | Edwards Sapien 3 26 |
| 1. Hancock II 29 | Edwards Sapien 3 26 |
| 1. Hancock II 33 | Edwards Sapien 3 29 |
| 1. Carpentier Edwards 27 | Edwards Sapien 3 26 |
| 1. Carpentier Edwards 29 | Edwards Sapien 3 29 |
| 1. Xenograft 31 | Edwards Sapien 3 29 |
| 1. Mosaic 27 | Edwards Sapien 3 26 |
